# Supplementary material for: The Hirudo Medicinalis Microbiome Is a Source of New Antimicrobial Peptides
Source: Int J Mol Sci. 2020 Sep 27;21(19):7141. doi: 10.3390/ijms21197141 (PMC7582656; doi:10.3390/ijms21197141)
Supplement: Supplementary file 1 [file ijms-21-07141-s001.pdf]

International Journal of *Molecular Sciences*

MDPI

The *Hirudo Medicinalis* Microbiome Is a Source of New Antimicrobial Peptides

Ekaterina Grafskaia [1,*], Elizaveta Pavlova [1,2], Vladislav V. Babenko [1], Ivan Latsis [1], Maja Malakhova [1], Victoria Lavrenova [1,3], Pavel Bashkirov [1], Dmitrii Belousov [4], Dmitry Klinov [1] and Vassili Lazarev [1,2,*]

1 Federal Research and Clinical Center of Physical Chemical Medicine of Federal Medical Biological Agency, Moscow 119435, Russia; grafskayacath@gmail.com (E.G.); lizapavlova6@gmail.com (E.P.); daniorerio34@gmail.com (V. B.); lacis.ivan@gmail.com (I.L.); maja_m@mail.ru (M.M.); lavrv1995@gmail.com (V.L.); pavel.bashkirov@niifhm.ru (P.B.); klinov.dmitry@mail.ru (D.K.)
2 Moscow Institute of Physics and Technology, 141700 Dolgoprudny, Moscow region 141701, Russia; lizapavlova6@gmail.com
3 Department of biochemistry, Faculty of Biology, Lomonosov Moscow State University, Moscow 119991, Russia; lavrv1995@gmail.com
4 Sechenov First Moscow State Medical University Sechenov University, Moscow 119991, Russia; belik2011@mail.ru
* Correspondence: grafskayacath@gmail.com (E.G.), lazarev@rcpcm.org (V.L.)

Received: 3 September 2020; Accepted: 25 September 2020; Published: 27 September 2020

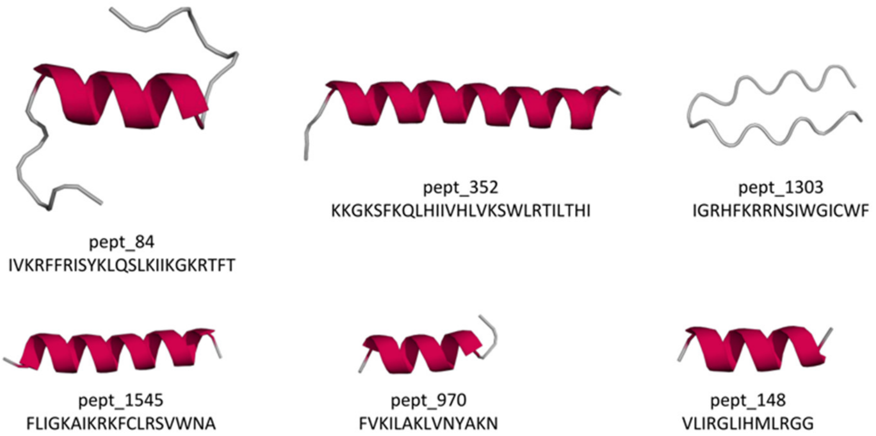

Figure S1. Predicted secondary structures of AMPs by the I–TASSER–MR server. The secondary structure region that adopts an α–helical conformation is indicated in red.

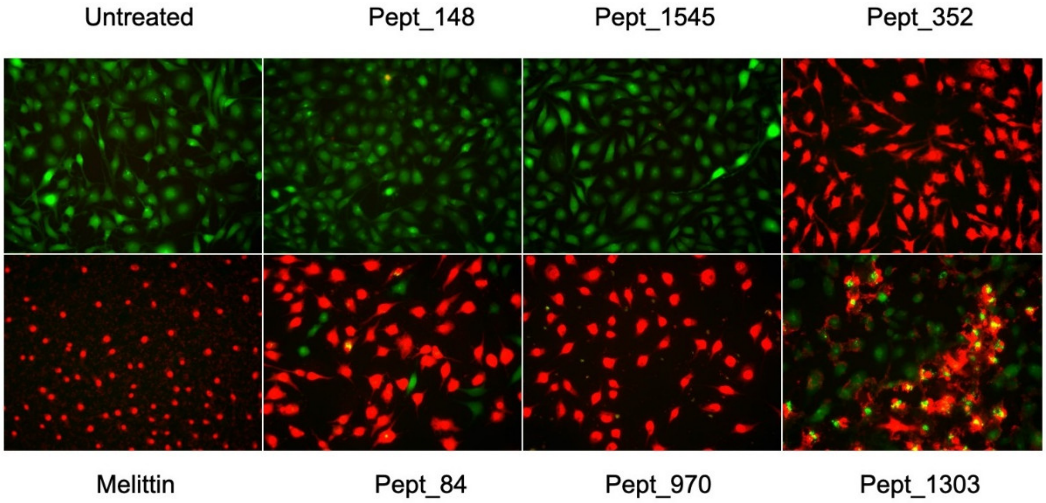

Figure S2. Effect of AMPs on the survival of McCoy cells. Cells were treated for 24 h with peptides at a final concentration equal to 4× MIC. After staining with calcein AM and ethidium homodimer–1, samples were analysed by fluorescence microscopy. The peptide melittin was used as a positive control. The negative control corresponds to the McCoy cells incubated without peptide (DMEM). The size of each individual frame is 485 μm× 366 μm.

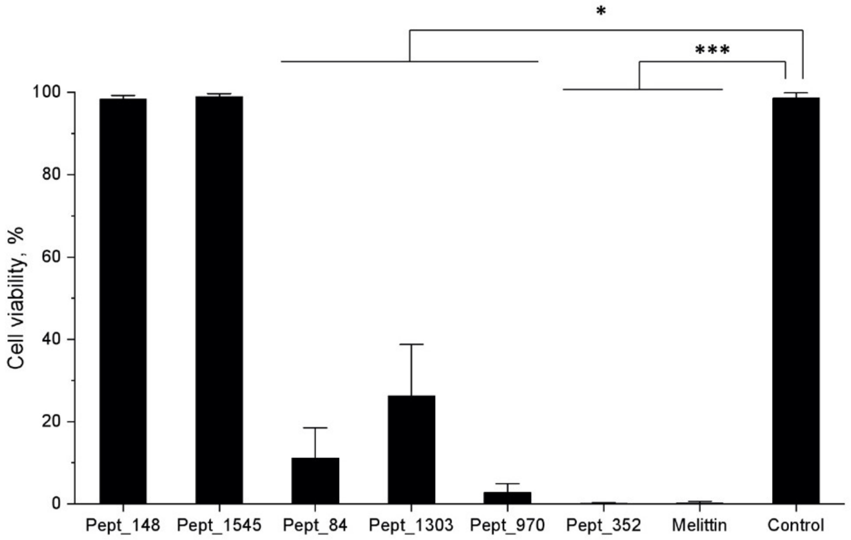

Figure S3. Viability of AMP–treated McCoy cells measured by LHD assay. Cells were treated for 24 h with peptides at a final concentration equal to 4× MIC. Melittin was used as a negative control. The positive control corresponds to untreated cells (Control). The values are represented as the mean ± SD (n = 3). Statistically significant differences between the control and experimental groups were determined by the non–parametric Kruskal–Wallis test, $^{*}p < 0.05$, $^{***}p < 0.005$.

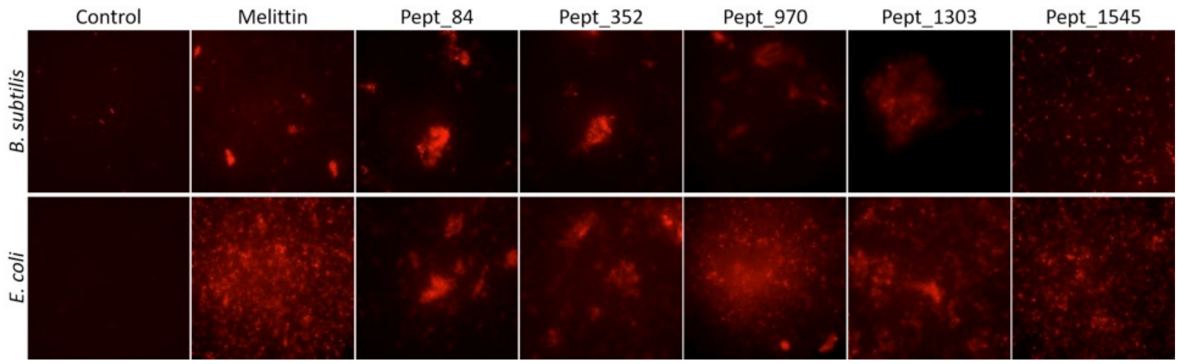

Figure S4. Effect of AMPs on the survival of bacterial cells after peptide treatment. *B. subtilis* (**top row)** and *E. coli* (**bottom row**) were incubated with peptides at a final concentration equal to 2× MIC at 37 °C for 1 h. After staining with PI, samples were analyzed by fluorescence microscopy. The peptide melittin was used as a positive control. The negative control corresponds to the bacteria incubated without peptide (MHB). The size of each individual frame is 222 μm× 222 μm.

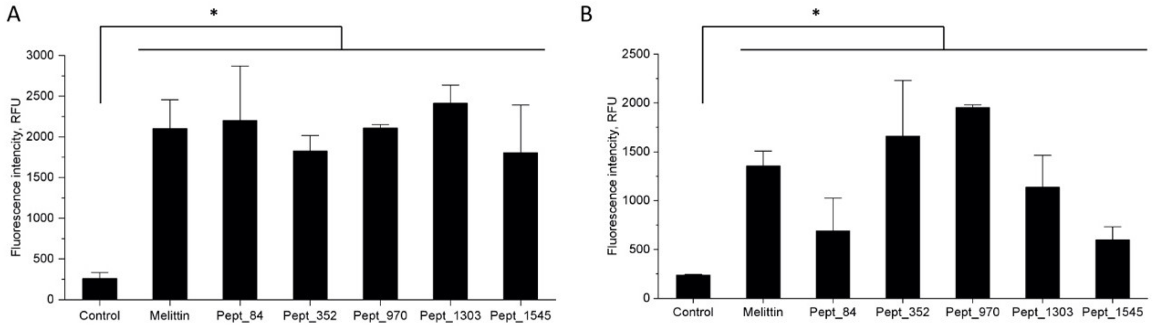

Figure S5. Effect of AMPs on the survival of bacterial cells after peptide treatment. *B. subtilis* (**A**) and *E. coli* (**B**) were incubated with peptides at a final concentration equal to 2× MIC at 37 °C for 1 h. After staining with PI, samples were analysed by fluorescence microscopy. The peptide melittin was used as a positive control. The negative control corresponds to the bacteria incubated without peptide (MHB). The column indicates the mean (±SD) fluorescence intensity for *B. subtilis* and *E. coli* strained with PI and treated with peptides and control cells. Statistically significant differences between the control and experimental groups were determined by the non–parametric Kruskal–Wallis test, *$p < 0.05$.

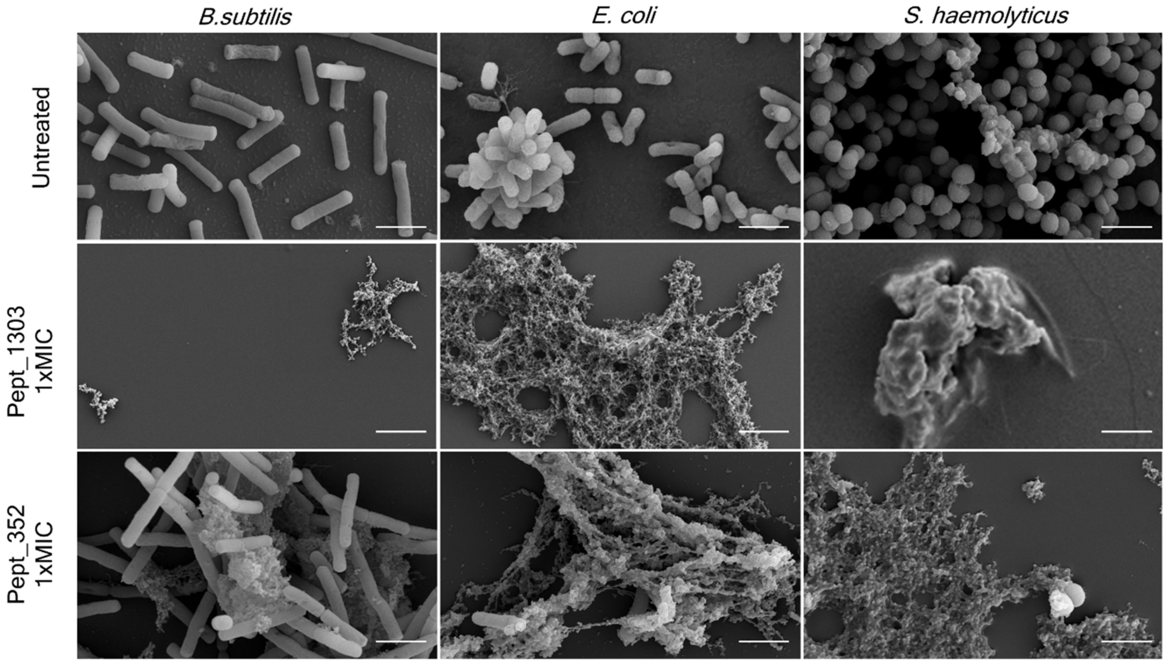

Figure S6. AMPs affect bacterial membrane integrity. Scanning electron microscopy (SEM) images of bacterial strains treated with peptides pept_352 and pept_1303. *B. subtilis* and *E. coli* were treated with peptides at a final concentration equal to 1/2× MIC and 1× MIC for 8 h, and *S. haemolyticus* was treated with peptides at a final concentration equal to 1/2× MIC and 1× MIC for 24 h. Scale bars: 2 μm.

Table S1. Data of AMP secondary structures based on the deconvolution analysis of CD spectra.

Peptide	Conditions	Percentage of each secondary structure				
		Helix	Antiparallel	Parallel	Turn	Disordered
pept_1303	aqueous solution	0.4±0.2	29.0±0.7	0.0±0.0	15.2±0.4	55.3±0.9
	buffer	0.8±0.1	30.3±0.3	0.0±0.0	15.0±0.1	53.9±0.1

	buffer + POPC liposomes	0.8±0.1	28.2±4.3	0.0±0.0	15.3±0.7	55.6±3.7
pept_148	aqueous solution	0.2±0.1	25.8±0.3	0.0±0.0	18.4±0.4	55.6±0.4
	buffer	0.9±0.1	21.5±2.3	0.0±0.0	19.1±0.8	58.6±1.7
	buffer + POPC liposomes	1.1±0.1	22.0±2.7	0.0±0.0	18.8±0.8	58.2±2.0
pept_970	aqueous solution	0.0±0.0	26.0±2.1	0.0±0.0	18.3±0.1	55.7±2.2
	buffer	0.0±0.0	23.6±0.9	0.0±0.0	17.7±0.4	58.5±1.8
	buffer + POPC liposomes	0.8±0.1	23.7±2.3	0.0±0.0	17.5±0.1	58.0±2.2
pept_1545	aqueous solution	0.0±0.0	20.9±0.7	0.0±0.0	18.0±0.3	61.0±3.6
	buffer	0.0±0.0	26.0±0.2	0.0±0.0	17.8±0.4	56.3±0.4
	buffer + POPC liposomes	0.0±0.0	25.7±0.6	0.0±0.0	17.7±0.2	56.3±0.4
pept_352	aqueous solution	2.6±0.2	25.2±3.5	0.0±0.0	16.4±1.0	55.9±2.8
	buffer	12.6±2.7	25.1±2.3	4.6±0.1	13.5±0.1	44.6±0.4
	buffer + POPC liposomes	17.1±0.5	24.2±0.2	5.0±0.1	12.5±0.1	41.2±0.4
pept_84	aqueous solution	0.0±0.0	26.7±0.5	0.0±0.0	17.8±0.1	55.5±0.4
	buffer	0.0±0.0	28.3±0.3	0.0±0.0	17.8±0.1	54.0±0.3
	buffer + PC liposomes	0.0±0.0	28.6±0.5	0.0±0.0	17.9±0.1	53.5±0.4
Melittin	aqueous solution	3.3±0.1	29.0±1.2	0.0±0.0	16.5±0.2	51.3±1.1
	buffer	4.3±0.3	28.1±0.8	0.0±0.0	16.3±0.2	51.3±0.6
	buffer + PC liposomes	11.5±0.8	21.5±4.9	6.8±0.9	14.4±0.6	45.9±2.7

Table S2. The physical-chemical characteristics of AMPs from the *H. medicinalis* microbiome.

Peptide	Amino acid sequence	Charge	Protein-binding potential (Boman index), kcal/mol	Hydrophobic ratio, %
pept_1303	IGRHFKRRNSIWGICWF	+4	2.14	47
pept_148	VLIRGLIHMLRGG	+2	0.05	53
pept_970	FVKILAKLVNYAKN	+3	0.04	57
pept_1545	FLIGKAIKRKFCLRSVWNA	+5	0.98	57
pept_352	KKGKSFKQLHIIVHLVKSWLRTILTHI	+6	0.75	44
pept_84	IVKRFFRISYKLQSLKIIKGKRTFT	+8	1.86	40
